# Supplementary material for: Establishment of Novel Neuroendocrine Carcinoma Patient-Derived Xenograft Models for Receptor Peptide-Targeted Therapy
Source: Cancers (Basel). 2022 Apr 10;14(8):1910. doi: 10.3390/cancers14081910 (PMC9033026; doi:10.3390/cancers14081910)
Supplement: Supplementary file 1 [file cancers-14-01910-s001.zip › cancers-1664107-supplementary.pdf]

Supplementary Table S1. List of curated mutations identified in NEC913 PDX tumors by exome sequencing.

Chr	Start	End	Ref	Alt	avsnp150	Func	Gene	ExonicFunc	AAChange
chr2	212652834	212652834	C	T	rs375361752	exonic	ERBB4	nonsynonymous SNV	ERBB4:NM_001042599:exon4:c.G472A:p.A158T,ERBB4:NM_005235:exon4:c.G472A:p.A158T
chr6	32188296	32188296	C	T	rs561687277	exonic	NOTCH4	nonsynonymous SNV	NOTCH4:NM_004557:exon6:c.G1045A:p.G349S
chr6	161533750	161533750	C	G	nan	exonic	MAP3K4	nonsynonymous SNV	MAP3K4:NM_001363582:exon24:c.C4408G:p.R1470G,MAP3K4:NM_006724:exon24:c.C4420G:p.R1474G,MAP3K4:NM_001301072:exon25:c.C4558G:p.R1520G,MAP3K4:NM_005922:exon25:c.C4570G:p.R1524G,MAP3K4:NM_001291958:exon26:c.C2929G:p.R977G
chr6	80717709	80717709	G	C	nan	exonic	TTK	nonsynonymous SNV	TTK:NM_001166691:exon3:c.G323C:p.S108T,TTK:NM_003318:exon3:c.G323C:p.S108T
chr10	6527143	6527143	G	A	rs2236379	exonic	PRKCQ	nonsynonymous SNV	PRKCQ:NM_001282645:exon9:c.C614T:p.P205L,PRKCQ:NM_001323266:exon9:c.C614T:p.P205L,PRKCQ:NM_001242413:exon10:c.C989T:p.P330L,PRKCQ:NM_001282644:exon10:c.C881T:p.P294L,PRKCQ:NM_001323265:exon10:c.C989T:p.P330L,PRKCQ:NM_001323267:exon10:c.C881T:p.P294L,PRKCQ:NM_006257:exon10:c.C989T:p.P330L
chr11	108168057	108168057	G	T	nan	exonic	ATM	nonsynonymous SNV	ATM:NM_000051:exon33:c.G4953T:p.L1651F,ATM:NM_001351834:exon34:c.G4953T:p.L1651F
chr11	62446348	62446348	G	-	nan	exonic	UBXN1	**frameshift deletion**	UBXN1:NM_001286077:exon1:c.48delC:p.R17Gfs*23,UBXN1:NM_001286078:exon1:c.48delC:p.R17Gfs*83,UBXN1:NM_015853:exon1:c.48delC:p.R17Gfs*23
chr13	48942704	48942704	-	CG	nan	exonic	RB1	**frameshift insertion**	RB1:NM_000321:exon11:c.1091_1092insCG:p.E364Dfs*4
chr13	48942705	48942705	-	GGT	nan	exonic	RB1	nonframeshift insertion	RB1:NM_000321:exon11:c.1092_1093insGGT:p.E364_E365insG
chr13	114782808	114782808	G	A	rs781295846	exonic	RASA3	**stopgain**	RASA3:NM_001320822:exon12:c.C1015T:p.R339X,RASA3:NM_007368:exon12:c.C1111T:p.R371X
chr17	7577022	7577022	G	A	rs121913344	exonic	TP53	**stopgain**	TP53:NM_001126115:exon4:c.C520T:p.R174X,TP53:NM_001126116:exon4:c.C520T:p.R174X,TP53:NM_001126117:exon4:c.C520T:p.R174X,TP53:NM_001276697:exon4:c.C439T:p.R147X,TP53:NM_001276698:exon4:c.C439T:p.R147X,TP53:NM_001276699:exon4:c.C439T:p.R147X,TP53:NM_001126118:exon7:c.C799T:p.R267X,TP53:NM_000546:exon8:c.C916T:p.R306X,TP53:NM_001126112:exon8:c.C916T:p.R306X,TP53:NM_001126113:exon8:c.C916T:p.R306X,TP53:NM_001126114:exon8:c.C916T:p.R306X,TP53:NM_001276695:exon8:c.C799T:p.R267X,TP53:NM_001276696:exon8:c.C799T:p.R267X,TP53:NM_001276760:exon8:c.C799T:p.R267X,TP53:NM_001276761:exon8:c.C799T:p.R267X
chr18	42456670	42456670	-	TCTT	rs3085861	exonic	SETBP1	**frameshift insertion**	SETBP1:NM_001130110:exon4:c.681_682insTCTT:p.T228Sfs*8

Supplementary Table S2. List of curated mutations identified in NEC1452 PDX tumors by exome sequencing.

Chr	Start	End	Ref	Alt	avsnp150	Func	Gene	ExonicFunc	AAChange
chr1	89835209	89835209	G	A	rs75966734	exonic	GBP6	nonsynonymous SNV	GBP6:NM_198460:exon3:c.G295A:p.E99K
chr2	183066203	183066203	C	T	rs369181374	exonic	PDE1A	nonsynonymous SNV	PDE1A:NM_001258314:exon9:c.G1034A:p.R345Q,PDE1A:NM_001258313:exon10:c.G1088A:p.R363Q,PDE1A:NM_001363871:exon10:c.G1088A:p.R363Q,PDE1A:NM_005019:exon10:c.G1136A:p.R379Q,PDE1A:NM_001003683:exon11:c.G1136A:p.R379Q,PDE1A:NM_001258312:exon11:c.G1148A:p.R383Q
chr4	1019055	1019056	CA	-	rs145808953	exonic	FGFRL1	**frameshift deletion**	FGFRL1:NM_021923:exon6:c.1435_1436del:p.H485Lfs*66,FGFRL1:NM_001004356:exon7:c.1435_1436del:p.H485Lfs*66,FGFRL1:NM_001004358:exon7:c.1435_1436del:p.H485Lfs*66,FGFRL1:NM_001370296:exon7:c.1435_1436del:p.H485Lfs*66
chr4	72897699	72897699	G	-	rs569440022	exonic	NPFFR2	**frameshift deletion**	NPFFR2:NM_004885:exon1:c.81delG:p.R29Gfs*35
chr5	112116592	112116592	C	T	rs587781392	exonic	APC	**stopgain**	APC:NM_001127511:exon5:c.C667T:p.R223X,APC:NM_001354897:exon5:c.C667T:p.R223X,APC:NM_001354900:exon5:c.C460T:p.R154X,APC:NM_001354901:exon5:c.C460T:p.R154X,APC:NM_001354902:exon5:c.C667T:p.R223X,APC:NM_001354905:exon5:c.C460T:p.R154X,APC:NM_000038:exon6:c.C637T:p.R213X,APC:NM_001354895:exon6:c.C637T:p.R213X,APC:NM_001354896:exon6:c.C637T:p.R213X,APC:NM_001354898:exon6:c.C562T:p.R188X,APC:NM_001354899:exon6:c.C637T:p.R213X,APC:NM_001354903:exon6:c.C637T:p.R213X,APC:NM_001354904:exon6:c.C562T:p.R188X,APC:NM_001127510:exon7:c.C637T:p.R213X
chr5	112151184	112151184	A	G	rs1064793022	intronic	APC	nan	nan
chr5	180043973	180043973	G	A	rs149033942	exonic	FLT4	nonsynonymous SNV	FLT4:NM_001354989:exon22:c.C3023T:p.P1008L,FLT4:NM_002020:exon22:c.C3023T:p.P1008L,FLT4:NM_182925:exon22:c.C3023T:p.P1008L
chr6	80717709	80717709	G	C	nan	exonic	TTK	nonsynonymous SNV	TTK:NM_001166691:exon3:c.G323C:p.S108T,TTK:NM_003318:exon3:c.G323C:p.S108T
chr6	80717710	80717710	T	A	nan	exonic	TTK	nonsynonymous SNV	TTK:NM_001166691:exon3:c.T324A:p.S108R,TTK:NM_003318:exon3:c.T324A:p.S108R
chr6	154567863	154567863	C	T	rs34427887	exonic	IPCEF1;OPRM	**stopgain**	OPRM1:NM_001008503:exon4:c.C1201T:p.R401X
chr7	97736519	97736521	GCT	-	rs776837018	exonic	LMTK2	nonframeshift deletion	LMTK2:NM_014916:exon1:c.30_32del:p.L16del
chr9	390478	390478	A	G	nan	exonic	DOCK8	nonsynonymous SNV	DOCK8:NM_001190458:exon22:c.A2582G:p.H861R,DOCK8:NM_001193536:exon23:c.A2678G:p.H893R,DOCK8:NM_203447:exon24:c.A2882G:p.H961R
chr9	139235526	139235526	C	A	rs59873903	exonic	GPSM1	**stopgain**	GPSM1:NM_015597:exon9:c.C1283A:p.S428X
chr10	103530307	103530307	T	A	nan	exonic	FGF8	nonsynonymous SNV	FGF8:NM_001206389:exon5:c.A202T:p.N68Y,FGF8:NM_006119:exon5:c.A427T:p.N143Y,FGF8:NM_033165:exon5:c.A394T:p.N132Y,FGF8:NM_033163:exon6:c.A514T:p.N172Y,FGF8:NM_033164:exon6:c.A481T:p.N161Y
chr13	48951086	48951086	G	-	nan	exonic	RB1	**frameshift deletion**	RB1:NM_000321:exon13:c.1248delG:p.R418Efs*2
chr17	7578212	7578212	G	A	rs397516436	exonic	TP53	**stopgain**	TP53:NM_001126115:exon2:c.C241T:p.R81X,TP53:NM_001126116:exon2:c.C241T:p.R81X,TP53:NM_001126117:exon2:c.C241T:p.R81X,TP53:NM_001276697:exon2:c.C160T:p.R54X,TP53:NM_001276698:exon2:c.C160T:p.R54X,TP53:NM_001276699:exon2:c.C160T:p.R54X,TP53:NM_001126118:exon5:c.C520T:p.R174X,TP53:NM_000546:exon6:c.C637T:p.R213X,TP53:NM_001126112:exon6:c.C637T:p.R213X,TP53:NM_001126113:exon6:c.C637T:p.R213X,TP53:NM_001126114:exon6:c.C637T:p.R213X,TP53:NM_001276695:exon6:c.C520T:p.R174X,TP53:NM_001276696:exon6:c.C520T:p.R174X,TP53:NM_001276760:exon6:c.C520T:p.R174X,TP53:NM_001276761:exon6:c.C520T:p.R174X
chr19	18499449	18499449	T	G	rs372120002	exonic	GDF15	nonsynonymous SNV	GDF15:NM_004864:exon2:c.T631G:p.C211G

Supplementary Table S3. Short tandem repeat analyses of NEC913 PDX tumor compared to patient periphery blood mononuclear cells (PBMCs).

Marker Name	NEC913 Blood PBMCs		NEC913 PDX Sample	
	NEC913 Blood PBMCs	Compared to DSMZ STR Database	NEC913 PDX Sample	Compared to NEC913 Blood PBMCs
AMEL	X, Y	No match	X	X, Y
CSF1P0	10, 12	No match	10	10, 12
D13S317	12	No match	12	12
D16S539	10, 11	No match	10	10, 11
D5S818	10, 12	No match	12	10, 12
D7S820	8, 11	No match	8, 11	8, 11
TH01	7, 9.3	No match	7, 9.3	7, 9.3
TPOX	8	No match	8	8
vWA	16, 18	No match	16, 18	16, 18
Identity Match	No match between NEC913 Blood PBMCs and other samples in DSMZ STR Database of IDEXX.		>80% match between NEC913PDX and NEC913 Blood PBMCs samples.	

Supplementary Table S4. Short tandem repeat analyses of NEC1452 PDX tumor.

Marker Name	NEC1452 PDX Sample	Compared to DSMZ STR Database
AMEL	X	No match
CSF1P0	10, 11	No match
D13S317	12	No match
D16S539	12	No match
D5S818	10, 11	No match
D7S820	9, 10	No match
TH01	9, 9.3	No match
TPOX	8, 10	No match
vWA	18	No match
Identity Match	No match between NEC1452 PDX sample and other samples in DSMZ STR Database of IDEXX.	
